# Behavioral risk factors in oncology patients: A matched case control study from Yemen

**DOI:** 10.1371/journal.pone.0329534

**Published:** 2025-08-07

**Authors:** Mansour Abdu Al-Taj, Arzaq Mohammed Al-Salahi, Asad Ali Al-Salami, Aya Lutf Aldhurafi, Essa Mohammed Ghaliah, Jalila Abdulsalam Al-Yarimi, Majdi Abdullah Al-Rosi, Rashed Hussien Ahmed, Sara Abdullah Al-Jrrash, Tasneem Fuad Al-Sabri

**Affiliations:** 1 Department of Community Medicine, Faculty of Medicine and Health Sciences, Sana’a University, Sana’a, Yemen; 2 Faculty of Medicine and Health Sciences, Sana’a University, Sana’a, Yemen; PLOS: Public Library of Science, ETHIOPIA

## Abstract

**Background:**

Cancer is a significant public health challenge globally, marked by the uncontrolled growth of abnormal cells that can lead to severe morbidity and mortality. Modifiable lifestyle factors significantly influence cancer risk. The study aimed to investigate the behavioral risk factors of cancer among Yemeni adults, highlighting the critical need for targeted prevention strategies.

**Methods:**

A matched case-control study design was used. It carried out at the National Center of Oncology in Sana’a, Yemen, and the largest referral center for cancer patients in Yemen. The study included 680 participants, consisting of 340 newly diagnosed cancer cases and 340 matched controls. Controls matched cases on age, sex, and place of resident. All subjects volunteered to participate and were personally interviewed using a structured questionnaire that covered socio-demographic, behaviors and dietary intake data. Crude odd ratios (COR) and adjusted odds ratios (AOR) were estimated using conditional logistic regression and the level of significance was set at p-value < 0.05.

**Results:**

Our analysis found that those who have any family member with a history of cancer (AOR = 2.84; 95% confidence interval (CI): 1.59–5.06), consuming bread or food made from white flour more than once a day (AOR = 2.21; 95% CI: 1.03–4.73), frequently consuming animal oil three to seven times per week (AOR = 3.43; 95% CI: 1.76–6.70), and consuming soft drinks three to seven times per week (AOR = 2.08; 95% CI: 1.08–4.01) were at higher risk of cancer. However, those who had no formal work but supporting family activities (AOR = 0.35; 95% CI: 0.15–0.79), consuming fruit frequently during its seasons (AOR = 0.25; 95% CI: 0.09–0.67), consuming coffee three to seven times (AOR = 0.36; 95% CI: 0.21–0.62) or one to two times per week (AOR = 0.44; 95% CI: 0.23–0.81) were less likely to have cancer. Additionally, no significant differences found between cases and controls regarding education level, smoking, chewing shamma, and frequent consumption of other food and beverages.

**Conclusion:**

Frequent consumption of bread or products made from white flour, animal fats, and soft drinks were statistically associated with cancer. On the contrary, moderate coffee consumption and frequent intake of seasonal fruits showed to be protected from cancer. Additionally, a family history of cancer was identified as a significant risk factor for developing the disease. To mitigate cancer risk in Yemen, implementing health education campaigns through media to promote awareness of these dietary influences are needed. Additionally, community initiatives should encourage healthier eating habits, emphasizing the importance of fresh fruits and moderate consumption of coffee, and reducing the intake of food made from white flour, animal fat and sugary beverages.

## Introduction

Cancer is characterized by the uncontrolled growth and spread of abnormal cells that attack neighboring parts of the body and spread to other organs, which may lead to a variety of life-threatening conditions [1–3]. The World Health Organization estimated approximately 20 million new cancer cases and 9.7 million cancer deaths in 2022, and that one in five people will develop cancer during their lifetime [4]. Additionally, a recent study estimated that between 2020 and 2050, cancer will cost the global economy $25.2 trillion [5]. The complexity of cancer arises not only from its diverse forms, but also from the many risk factors that contribute to its development. Cancer cases are expected to increase by 77% in 2050 compared to 2022, reaching 35 million new cases per year [4] because of the changes in risk factors, which are usually related to population aging and growth, socio-economic status of people, and environmental factors [4].

The risk of cancer has been widely studied, and many studies have revealed the association of lifestyle, environmental and genetic influences on the development of different types of cancer. Lifestyle choices such as smoking [6–8], alcohol consumption [8–10], poor diet [11–13], and physical inactivity [14–16] increase the risk of cancer. Many environmental factors, such as exposure to radiation [17,18], air pollution [19–21], and certain chemicals [22,23], contribute to the risk of developing cancer. In addition, genetic predispositions are of great importance, as individuals with a family history of cancer may carry genetic mutations that increase their risk [24–26]. However, due to the rapid changes in behaviors and lifestyles, technological advancements, and disparities in food production and quality control that are strong in high-income countries and weak in low-income countries, there is a need to continue assessing the risk factors related to cancer development, particularly in low-income nations.

Cancer is an emerging public health challenge in Yemen, with the incidence of various types of cancer is increasing due to ongoing conflict and limited healthcare resources. In 2020, the World Health Organization reported that around 35,000 Yemenis were living with cancer, with over 11,000 new cases diagnosed each year [27]. The closure of many cancer clinics, caused by shortages of staff, medications, and equipment, has led to long waiting times and limited access to essential treatment, leaving thousands facing a death sentence [27]. The risk factors for cancer in Yemen have not been studied well. A study carried out in Hadramout governorate reported that divorced women, women who had never breastfed a child, having hypertension, family history of malignancy were at higher risk of breast cancer [28]. Further, chewing tobacco and frequent consumption of white bread found to be associated with gastric cancer [29]. The lack of data on cancer risk factors in Yemen hinders the development of effective prevention strategies. This study aims to address this critical gap by identifying behavioral risk factors associated with cancer development. Additionally, the findings will guide decision-makers in formulating healthcare policies and generate hypotheses for further research.

## Methods

### Study designs, setting, and population

A matched case-control study design was used, with a ratio 1:1. The cases were obtained from the Oncology Center in Sanaa city, which is the largest Oncology Center in Yemen and equipped with advanced technology, attracting cancer patients from all governorates. The study included patients who attended the Oncology Center in Sanaa city during the study period, with diagnoses confirmed by histopathology for any type of cancer. Only newly diagnosed cases within the six months prior to the interview were included. Patients with any type of old cancer, or with metastasis were excluded. The main types of cancer identified in this study include breast cancer (C50), oral cancer (C00-C14), cancer of the digestive organs (C15-C24), cancer of the female genital organs (C51-C58), Hodgkin lymphoma (C81), and Non-Hodkin lymphoma (C85).

The control group consisted of relatives of non-cancer patients admitted to one of three main hospitals in Sanaa city (Al-Thawra, Al-Jomhory, and Al-Kuwait hospitals), who self-reported not being diagnosed with any type of cancer. Individuals who had been diagnosed with cancer, were cured of cancer, or had any history of tumors were not selected as controls. Controls were matched to the cases based on age (± 2 years), sex, place of residence (rural or urban), and the governorate from which the cases originated. Postmenopausal women with breast cancer were matched with postmenopausal controls. Controls were selected from relatives of patients attending these facilities for feasibility, as it was challenging to obtain controls from the field. The cases came from various governorates in Yemen, where access is difficult due to security issues, poor road conditions, and financial and time constraints. Both cases and controls were aged 18 years and above.

### Sample size

With an alpha level of 0.05 and a power of 0.80, assuming a smoking prevalence of 0.50 among the control group and an odds ratio of 2, along with a case-control ratio of 1 and a correlation of smoking between cases and controls of 0.5, the calculated required sample size was 270 cases. After adjusting for a 20% nonresponse rate, the sample size increased to approximately 337, resulting in a final total of about 340 cases and similar number of controls.

### Data collection

First, data on the diagnosis of cancer, including type, size, and stage was obtained from the patients’ medical files by trained medical personnel. Subsequently, face-to-face interviews with cases were conducted using a structured questionnaire. We initially collected data from the cases until the required sample size was reached, and then collected data from the control group. Data collection took place from June to August 2024. The questionnaire consisted of questions regarding exposures, including demographic factors, behaviors, family history of cancers, and dietary habits.

### Study variables

The main outcome of this study was cancer. Cancer incident cases were identified and coded according to the International Classification of Diseases, 10th Edition (ICD-10). Independent variables included education level (no education, primary, secondary, university or above) and marital status (single, currently married, or formerly married). Respondents self-reported their main type of work, which was categorized into five groups: employed, farming, daily wages, supporting families in collecting water, wood for cooking, or grass for animals, and housewives. Respondents reported their smoking status as one of three categories: current smokers, former smokers, or have never smoked. Respondents were asked about the duration of their smoking habits, and then we reclassified as less than a year, one to five years, or more than five years. Additionally, respondents were asked whether they chew shamma (shamma is a type of powdered tobacco that users place in the mouth as a small wad, either in the labial vestibule or between the lower lip and gums in the buccal area) [30], and their responses were classified as yes or no.

We used the Diet History Questionnaire II from the Canadian National Cancer Institute, which is designed to assess dietary intake over the past year. This tool was adapted to fit the Yemeni context prior to its implementation [31]. A list of 130 food types and beverages commonly consumed in Yemen was created by the researchers and presented to respondents. These food types were then categorized into 11 food groups based on the Food and Agriculture Organization classifications for food groups and subgroups [32], with modifications made to suit the Yemeni context. These groups include: 1. Bread or food made from white flour, 2. Whole grains, 3. Pulses, nuts and seeds, 4. Milk and milk products, 5. Eggs, 6. Fish, 7. Red meat (bovine, goat, and sheep), 8. Poultry (chicken), 9. Vegetables (leafy and non-leafy vegetables), 10. Fruit, and 11. Butters and oils of animal origin. Beverages are classified into four groups: coffee, red tea, soft drinks and fresh fruit and vegetable juices. Cases were instructed to answer based on their most common and typical habits that best represented their lifestyle from the one-year period preceding cancer detection, while controls were asked about their habits from the period of the interview to the preceding year. Respondents were requested to recall their consumption frequency for each food type. Responses were coded as follows: 1. never or rare for those who reported less than one time per month, 2. more than one time a day, 3. three to seven times per week, 4. one to two times per week, 5. one to three times per month. For fruit, one category was added for those consumed frequently in season.

### Data analysis

Stata version 16 was used for data analysis. Categorical variables were described using proportions. Conditional logistic regression was fitted to the data to allow for matching. In the bivariate analysis, COR was estimated and AOR was computed in multivariable analysis. Only variables with a p-value below 0.05 in the bivariate analysis were adjusted and included in the multivariable analysis. P-value was determined using the likelihood ratio test (LRT) and the level of significance was set at < 0.05. Multicollinearity between variables was assessed by checking the changes in the direction of effect and standard errors from crude to multivariable analysis. No collinearity was detected.

### Ethical Considerations

The study received approval from the ethical committee of faculty of medicine and health science, Sanaa University. Permissions were granted by the National Oncology Center, as well as Al-Thawra, Al-Jomhory, and Al-Kuwait hospitals prior to conducting the research. Verbal informed consent was obtained from respondents due to the high illiteracy rates. This consent was collected in the presence of a witness prior to interviews. The ethical committee approved this consent procedure, and confidentiality of the data was assured and maintained.

## Results

The analysis included 340 cases and 340 controls. Among the cases, 87 (25.6%) had breast cancer, 74 (21.8%) were diagnosed with cancer of the digestive organs, and 64 (18.8%) had oral cancer. The mean age of the cases was 45.6 years ± 14.5 standard deviation, and the mean age of the controls was 44.3 years ± 14.1 standard deviation. More than half of both the cases and controls had no formal education and 10.3% of cases and 8.8% of controls were employed. Around 60.0% of cases and controls were never smokers, while 22.4% of cases and 15.0% of controls reported chewing shamma. Around a quarter of cases and less than 15.0% of control reported a family history of cancer (Table 1).

**Table 1:** Distribution of cases and controls by respondent characteristics, behaviours and family history of cancer.

Variable	Cases		Controls		COR (95% CI)	P.value
	n	%	n	%		
Age of respondents (Mean ± SD)	45.6 ± 14.5		44.3 ± 14.1			
Sex of respondents						
Male	145	42.6	145	42.6		
Female	195	57.4	195	57.4		
Type of cancer						
Breast	87	25.6				
Digestive organs	74	21.8				
Oral	64	18.8				
Female genital organs	26	7.6				
Hodgkin lymphoma	21	6.2				
Non-Hodgkin lymphoma	33	9.7				
Others	35	10.3				
**Education level**						
No education	183	53.8	178	52.3	Ref.	0.369
Primary	53	15.6	64	18.8	0.80 (0.51-1.26)	
Secondary	68	20.0	58	17.1	1.11 (0.69-1.78)	
University and above	36	10.6	40	11.8	0.86 (0.49-1.49)	
**Work status**						
Employed	35	10.3	30	8.8	Ref.	0.001
Farmer	50	14.7	39	11.5	1.32 (0.63-2.79)	
Daily wages	51	15.0	47	13.8	0.98 (0.52-1.84)	
Supporting families	78	22.9	124	36.5	0.50 (0.28-0.91)	
Housewives	126	37.01	100	29.4	1.14 (0.60-2.17)	
**Smoking status**						
Current	85	25.0	85	25.0	Ref.	0.434
Former	57	16.8	38	11.2	1.53 (0.89-2.64)	
Never	198	58.2	217	63.8	0.90 (0.60-1.34)	
**Period of smoking in year**						
<one year	204	60.0	217	63.8	Ref.	0.213
One to five years	22	6.5	29	8.5	0.81 (0.44-1.47)	
> five years	114	33.5	94	27.7	1.38 (95-2.01)	
**Chewing shamma**						
No	264	77.6	289	85.0	Ref.	0.007
Yes	76	22.4	51	15.0	1.86 (1.18-2.92)	
**Family history of Cancer**						
No	249	73.2	291	85.6	Ref.	< 0.001
Yes	91	26.8	49	14.4	2.31 (1.53-3.50)	

n, number; %, percentage; COR, crude odds ratio; CI, confidence interval; SD, standard deviation; Ref., reference group.

In the study, 43.2% of the case group and 21.8% of the control group reported consuming food or bread made from white flour more than once per day. Approximately 30.0% of participants in both groups received pulse, nuts, and seed-containing foods three to seven times per week, while around two-thirds consumed vegetables three to seven times per week. Regarding fruit consumption, approximately 45.0% of both groups reported eating fruit one to two times per week. Furthermore, around 30.0% of participants in both groups consumed chicken three to seven times per week. Meat consumption was reported by 25.6% of cases and 18.2% of controls one to two times per week, while 20.6% of cases and 14.1% of controls reported consuming fish within the same frequency. Dairy consumption was high, with approximately 60.0% of both cases and controls consuming milk or dairy products three to seven times per week. In contrast, only 22.3% of cases and 13.8% of controls reported consuming eggs within the same frequency (Table 2).

**Table 2:** Distribution of cases and controls by food frequency.

Food intake	Cases		Control		COR (95% CI)	P.value
	n	%	n	%		
**Bread or food made from white flour**						
Rare or never	38	11.2	55	16.2	Ref.	< 0.001
> 1 times per day	147	43.2	74	21.8	3.19 (1.87-5.45)	
3 to 7 times per week	88	25.9	111	32.6	1.27 (0.76-2.11)	
1 to 2 times per week	36	10.6	56	16.5	0.99 (0.54-1.85)	
1 to 3 times per month	31	9.1	44	12.9	1.06 (0.56-1.98)	
**Whole grains**						
> 1 times per day	109	32.1	93	27.3	Ref.	0.086
3 to 7 times per week	170	50.0	193	56.8	0.74 (0.51-1.06)	
1 to 2 times per week	61	17.9	54	15.9	0.93 (0.57-1.52)	
**Pulses, nuts, and seed**						
Rare or never	47	13.8	73	21.5	Ref.	0.004
3 to 7 times per week	112	32.9	105	30.9	1.72 (1.08-2.73)	
1 to 2 times per week	127	37.4	97	28.5	2.09 (1.32-3.30)	
1 to 3 times per month	54	15.9	65	19.1	1.25 (0.77-2.03)	
**Vegetables**						
> 1 times per day	102	30.0	66	19.4	Ref.	0.001
3 to 7 times per week	199	58.5	217	63.8	0.55 (0.37-0.83)	
1 to 2 times per week	39	11.5	57	16.8	0.43 (0.25-0.72)	
**Fruit**						
3 to 7 times per week	58	17.1	55	16.2	Ref.	0.002
1 to 2 times per week	162	47.6	147	43.2	1.13 (0.70-1.84)	
1 to 3 times per month	99	29.1	85	25.0	1.20 (0.72-2.00)	
Frequently during the season	21	6.2	53	15.6	0.38 (0.20-0.74)	
**Chicken**						
3 to 7 times per week	110	32.4	107	31.5	Ref.	0.139
1 to 2 times per week	170	50.0	154	45.3	1.06 (0.72-1.53)	
1 to 3 times per month	60	17.6	79	23.2	0.68 (0.42-1.12)	
**Meat**						
Rare or never	170	50.0	204	60.0	Ref.	0.043
1 to 2 times per week	87	25.6	62	18.2	1.77 (1.17-2.65)	
1to 3 times per month	83	24.4	74	21.8	1.38 (0.93-2.05)	
**Fish**						
Rare or never	131	38.5	187	55.0	Ref.	< 0.001
3 to 7 times per week	36	10.6	16	4.7	4.06 (2.05-8.04)	
1 to 2 times per week	70	20.6	48	14.1	2.49 (1.56-3.98)	
1 to 3 times per month	103	30.3	89	26.2	1.80 (1.22-2.65)	
**Milk and milk products**						
3 to 7 times per week	214	62.9	206	60.6	Ref.	0.584
1 to 2 times per week	82	24.1	80	23.5	0.98 (0.68-1.41)	
1 to 3 times per month	44	12.9	54	15.9	0.78 (0.49-1.22)	
**Eggs**						
Rare or never	39	11.5	55	16.2	Ref.	< 0.001
3 to 7 times per week	76	22.3	47	13.8	2.53 (1.40-4.58)	
1 to 2 times per week	156	45.9	130	38.2	1.89 (1.11-3.22)	
1 to 3 times per month	69	20.3	108	31.8	0.96 (0.56-1.66)	
**Butters and oils of animal origin**						
Rare or never	163	47.9	210	61.8	Ref.	< 0.001
3 to 7 times per week	79	23.2	44	12.9	2.66 (1.67-4.25)	
1 to 2 times per week	62	18.2	47	13.8	1.86 (1.18-2.92)	
1 to 3 times per month	36	10.6	39	11.5	1.31 (0.79-2.17)	

n, number; %, percentage; COR, crude odds ratio; CI, confidence interval; Ref., reference group.

A small percentage of both the cases (12.1%) and the control group (3.2%) reported consuming coffee more than one time per day. Conversely, over 55.0% in both groups consumed red tea more than one time per daily. Additionally, 21.2% of the cases and 11.8% of the controls reported consuming soft drinks three to seven times a week (Table 3).

**Table 3:** Distribution of cases and controls by beverage frequency.

Beverage intake	Cases		Control		COR (95% CI)	P.value
	n	%	n	%		
**Coffee**						
Rare or never	150	44.1	137	40.3	Ref.	< 0.001
> 1 times per day	41	12.1	11	3.2	3.61 (1.71-7.62)	
3 to 7 times per week	83	24.4	125	36.8	0.61 (0.42-0.88)	
1 to 2 times per week	66	19.4	67	19.7	0.90 (0.59-1.39)	
**Red tea**						
> 1 times per day	196	57.7	188	55.3	Ref.	0.223
3 to 7 times per week	99	29.1	113	33.2	0.84 (0.60-1.18)	
1 to 2 times per week	45	13.2	39	11.5	1.10 (0.69-1.76)	
**Soft drinks**						
Rare or never	206	60.6	229	67.3	Ref.	0.001
3 to 7 times per week	72	21.2	40	11.8	2.23 (1.38-3.60)	
1 to 3 times per month	62	18.2	71	20.9	1.00 (0.65-1.54)	
**Fresh fruit and vegetable juices**						
Rare or never	125	36.8	160	47.1	Ref.	0.018
1 to 2 times per week	89	26.2	72	21.2	1.80 (1.15-2.80)	
1 to 3 times per month	126	37.0	108	31.7	1.57 (1.09-2.25)	

n, number; %, percentage; COR, crude odds ratio; CI, confidence interval; Ref., reference group.

In the bivariate analysis, those who were not formally employed but supported their families were 50% less likely to have cancer compared to those who were employed (COR = 0.50; 95% CI: 0.28–0.91). However, having a family history of cancer (COR = 2.31; 95% CI: 1.53–3.50), and chewing shamma (COR = 1.86; 95% CI: 1.18–2.92) were associated with cancer (Table 1). Individuals who consumed bread or food made from white flour more than once per day (COR = 3.19; 95% CI: 1.87–5.45), as well as those consuming pulses three to seven times (COR = 1.72; 95% CI: 1.08–2.73) or one to two times per week (COR = 2.09; 95% CI: 1.32–3.30), exhibited a higher risk of cancer compared to those who consumed these foods rarely or never. Additionally, participants who consumed meat one to two times per week (COR = 1.77; 95% CI: 1.17–2.65), fish at any frequency, eggs three to seven times per week (COR = 2.53; 95% CI: 1.40–4.58) or one to two times per week (COR = 1.89; 95% CI: 1.11–3.22), and animal oil either three to seven times (COR = 2.66; 95% CI: 1.67–4.25) or one to two times per week (COR = 1.86; 95% CI: 1.18–2.92) were also found to be at an increased risk of cancer compared to those with infrequent or no consumption of these items (Table 2). Compare to those who rarely or never consumed coffee, those who consumed it more than one times per day were at higher risk of developing cancer (COR = 3.61; 95% CI: 1.71–7.62) while those who consumed it three to seven times (COR = 0.61; 95% CI: 0.42–0.88) were significantly associated with lower risk of cancer. Consuming soft drinks three to seven times a week doubled the risk of cancer (COR = 2.23; 95% CI: 1.38–3.60) (Table 3).

Table 4 presents the variables that were significantly associated with cancer in the multivariable analysis, and all findings from the multivariable analysis can be found in S1 Table. Those who do not have a job but supporting family activities (AOR = 0.35; 95% CI: 0.15–0.79), consuming fruit during season (AOR = 0.25; 95% CI: 0.09–0.67), consuming coffee three to seven time a week (AOR = 0.36; 95% CI: 0.21–0.62) or one to two times a week (AOR = 0.44; 95% CI: 0.23–0.81), were more likely to be protected from cancer while those who have any family member with a history of cancer (AOR = 2.84; 95% CI: 1.59–5.06), those who consuming food or bread made from the white flour more than once a day (AOR = 2.21; 95% CI: 1.03–4.73), those consuming animal oil more than once a day (AOR = 3.43; 95% CI: 1.76–6.70), and those consuming soft drinks three to seven times a week remained at higher risk of developing cancer (AOR = 2.08; 95% CI: 1.08–4.01).

**Table 4:** Multivariable analysis for the risk factors of cancer in Yemen.

Adjusted Variables	AOR (95% CI)	P.value
**Respondent work**		
employed	Ref.	< 0.001
Farmer	1.36 (0.51-3.61)	
Daily wages	0.66 (0.29-1.52)	
Supporting families	0.35 (0.15-0.79)	
Housewives	1.57 (0.64-3.86)	
**Family history of Cancer**		
No	Ref.	< 0.001
Yes	2.84 (1.59-5.06)	
**Frequency of consuming bread or food made from white flour**		
Rare or never	Ref.	0.005
> 1 times per day	2.21 (1.03-4.73)	
3 to 7 times per week	1.27 (0.61-2.66)	
1 to 2 times per week	0.68 (0.28-1.62)	
1 to 3 times per month	0.76 (0.32-1.83)	
**Frequency of consuming fruit**		
3 to 7 times per week	Ref.	< 0.001
1 to 2 times per week	0.87 (0.44-1.73)	
1 to 3 times per month	1.51 (0.71-3.19)	
Frequently during the season	0.25 (0.09-0.67)	
**Frequency of consuming butters and oils of animal origin**		
Rare or never	Ref.	0.002
3 to 7 times per week	3.43 (1.76-6.70)	
1 to 2 times per week	1.51 (0.79-2.87)	
1 to 3 times per month	1.50 (0.72-3.12)	
**Frequency of consuming coffee**		
Rare or never	Ref.	< 0.001
> 1 times per day	1.62 (0.60-4.35)	
3 to 7 times per week	0.36 (0.21-0.62)	
1 to 2 times per week	0.44 (0.23-0.81)	
**Frequency of consuming soft drinks**		
Rare or never	Ref.	0.005
3 to 7 times per week	2.08 (1.08-4.01)	
1 to 3 times per month	0.64 (0.34-1.20)	

n, number; %, percentage; AOR, adjusted odds ratio; CI, confidence interval; Ref., reference group.

## Discussion

This study revealed that work status, familial cancer history, and some dietary habits serve as significant predictors of cancer among Yemenis. Among the respondents and household characteristics, only those who were unemployed but engaged in supportive activities such as collecting water, gathering wood for cooking, or tending to livestock demonstrated a lower risk of cancer compared to salaried employment. While salaried workers often have access to better healthcare and resources, they may also experience higher levels of stress and sedentary lifestyles associated with traditional office jobs. In contrast, the physically demanding nature of tasks like collecting water and tending to livestock may promote greater physical activity and social interaction, which can enhance overall well-being. A recent study from Switzerland found that female building caretakers and cleaners had a reduced incidence of breast cancer (SIR = 0.69, 95% CI: 0.59–0.81) compared to legal professionals (SIR = 1.68, 95% CI: 1.27–2.23), social science workers (SIR = 1.29, 95% CI: 1.12–1.49), and certain office workers (SIR = 1.14, 95% CI: 1.09–1.20) [33].

Having a family history of cancer is a significant risk factor for developing the disease. A recent study demonstrated the association between genetic predisposition and the risk of early-onset total cancer [34]. In the bivariate analysis, chewing shamma was associated with cancer risk, but this association was not significant in the multivariable analysis. This may be due to the mediation by other factors such as genetics. A previous case-control study conducted in Sana’a, Yemen, found that individuals who chewed shamma were 4.4 times more likely to develop gastric cancer than those who did not [29]. In this study neither smoking status nor duration of smoking were associated with cancer and this contradicted several studies that reported the association between smoking status and cancers [35,36]. The lack of association between the smoking status and cancer may be due to the high number of respondents in both groups who were not smokers.

We found that consumption of bread or food made from white flour was significantly associated with increased odds of cancer. Consuming foods made from white flour often have a lower content of fiber [37], and a high glycemic index [38], causing rapid spikes in blood sugar and insulin levels. Chronic high insulin levels can promote inflammation and cellular proliferation, creating an environment conducive to cancer development [39]. Our findings indicated that fruit intake in seasons have a protective effect against cancer is supported by the nutritional profile of fruits, which are rich in vitamins, minerals, antioxidants, and dietary fiber [40,41]. Consuming fruit during its seasons, when they are likely to be fresher and contain fewer pesticide residues or chemical substances used in rapid growth, may enhance their health benefits. Our finding indicates that individuals who consume animal oil through cooking or as a direct addition to their meals three to seven times per week show a higher risk of cancer compared to those who consume it rarely or never. This is in lines with other reports from different contexts [42]. Animal fats are often high in saturated fatty acids, which have been linked to increased levels of cholesterol and inflammation in the body, two factors that may contribute to cancer development. In the multivariable analysis, we found that none of the other food groups demonstrated a significant association with cancer. This may be due to the fact that the effects of dietary patterns may be influenced by other lifestyle factors, such as physical activity and genetic predispositions.

Among beverages, we found that coffee consumption, defined as three to seven times a week or one to two times a week, was associated with a reduced risk of cancer. Several studies and meta-analysis have reported protective associations of coffee with various cancer types, including colorectal [43–46], prostate [47], ovarian [48], and breast [49] cancers. On the other hand, other studies and meta-analyses found that consuming coffee was associated with an increased risk of lung [50], ovarian [51], and digestive system [52]. Coffee is a complex mixture of over 1,000 bioactive ingredients, including caffeine, and chlorogenic acids, which are important to exhibit anticancer potential, particularly due to the rich polyphenol content that provides antioxidant and anti-inflammatory effects that can protect against cellular damage [53,54]. Although the relationship between coffee consumption and cancer risk continues to be a subject of debate, our study provides a hypothesis for further experimental or cohort studies to investigate the role of consuming coffee three to seven times per week and or one to two times per week in reducing the risk of developing cancer. Frequent consumption soft drinks (three to seven times per week) was associated with cancer risk. Soft drinks are typically high in added sugars [55]. High sugar intake is linked to obesity [55], which is a significant risk factor for various cancers [56–58].

This study faced potential recall bias, as many questions required participants to report on their habits over the past year, with some inquiries extending to a lifetime history. Additionally, due to the case-control design, cases may have been more likely to remember details than controls. There is also a possibility that some controls may have had undiagnosed cancer. However, since the incidence of cancer is very low, this is unlikely to have significantly impacted our findings. Some factors, such as exercise, stress, and body mass index, were not addressed by this study. Therefore, further research with a larger sample size and well-designed cohorts is necessary to explore the relationships between these factors and cancer. Additionally, it is recommended that future studies investigate risk factors associated with specific common types of cancer, such as breast, colorectal, and oral cancer. It is important to emphasize that when interpreting the results of this study, one should consider that it focuses solely on the frequency of consumption rather than the quantity.

## Conclusion

Frequent consumption of bread and foods made from white flour, soft drinks, and animal fats is linked to an increased risk of developing cancer. Conversely, seasonal intake of fruits and moderate coffee consumption (one to two times, or three to seven times per week) provides protective benefits. To mitigate cancer risk in Yemen, it is essential to implement health education campaigns through media to raise awareness about these dietary influences. Community initiatives should promote healthier eating habits by emphasizing the importance of fruits while encouraging a reduction in the intake of processed foods and sugary beverages. Additionally, further research is needed to explore the impact of other factors, such as exercise, body mass index, and stress, employing designs like cohort studies to gain deeper understandings into their relationships with cancer risk.

## Supporting information

S1 Table Multivariable analysis for the risk factors of cancer in Yemen. (PDF)

S1 Dataset Research related data (XLSX)
