# Supplementary material for: Behavioral risk factors in oncology patients: A matched case control study from Yemen
Source: PLoS One. 2025 Aug 7;20(8):e0329534. doi: 10.1371/journal.pone.0329534 (PMC12331032; doi:10.1371/journal.pone.0329534)
Supplement: S1 Table — (PDF) [file pone.0329534.s001.pdf]

Table 1. S 1 Multivariable analysis for the risk factors of cancer in Yemen

Variables	AOR (95% CI)	LRT
**Respondent work**		
employed	Ref.	< 0.001
Farmer	1.36 (0.51-3.61)	
Daily wages	0.66 (0.29-1.52)	
Supporting families	0.35 (0.15-0.79)	
Housewives	1.57 (0.64-3.86)	
**Family history of Cancer**		
No	Ref.	< 0.001
Yes	2.84 (1.59-5.06)	
**Chewing shamma**		
No		0.098
Yes	1.65 (0.90-3.03)	
**Frequency of consuming bread or food made from white**		
Rare or never	Ref.	0.005
> 1 times per day	2.21 (1.03-4.73)	
3 to 7 times per week	1.27 (0.61-2.66)	
1 to 2 times per week	0.68 (0.28-1.62)	
1 to 3 times per month	0.76 (0.32-1.83)	
**Frequency of consuming fruit**		
3 to 7 times per week	Ref.	< 0.001
1 to 2 times per week	0.87 (0.44-1.73)	
1 to 3 times per month	1.51 (0.71-3.19)	
Frequently during the season	0.25 (0.09-0.67)	
**Frequency of consuming meat**		
Rare or never	Ref.	0.896
1 to 2 times per week	1.04 (0.56-1.91)	
1 to 3 times per month	1.15 (0.64-2.07)	
**Frequency of consuming Fish**		
Rare or never	Ref.	0.169
3 to 7 times per week	2.54 (0.97-6.64)	
1 to 2 times per week	1.58 (0.79-3.15)	
1 to 3 times per month	1.59 (0.91-2.78)	
**Frequency of consuming Pulses**		
Rare or never	Ref.	0.136
3 to 7 times per week	0.97 (0.47-2.03)	
1 to 2 times per week	1.56 (0.77-3.18)	
1 to 3 times per month	0.76 (0.37-1.56)	
**Frequency of consuming vegetables**		
> 1 times per day	Ref.	0.180
3 to 7 times per week	0.59 (0.33-1.06)	
1 to 2 times per week	0.80 (0.37-1.74)	
**Frequency of consuming eggs**		

Rare or never	Ref.	0.222
3 to 7 times per week	1.37 (0.60-3.14)	
1 to 2 times per week	1.15 (0.55-2.40)	
1 to 3 times per month	0.69 (0.33-1.46)	
**Frequency of consuming butters and oils of animal origin**		
Rare or never	Ref.	0.002
3 to 7 times per week	3.43 (1.76-6.70)	
1 to 2 times per week	1.51 (0.79-2.87)	
1 to 3 times per month	1.50 (0.72-3.12)	
**Frequency of consuming Coffee**		
Rare or never	Ref.	< 0.001
> 1 times per day	1.62 (0.60-4.35)	
3 to 7 times per week	0.36 (0.21-0.62)	
1 to 2 times per week	0.44 (0.23-0.81)	
**Frequency of consuming Soft drinks**		
Rare or never	Ref.	0.005
3 to 7 times per week	2.08 (1.08-4.01)	
1 to 3 times per month	0.64 (0.34-1.20)	
**Frequency of consuming fresh fruit and vegetable juices**		
Rare or never	Ref	0.817
1 to 2 times per week	0.95 (0.49-1.86)	
1 to 3 times per month	1.13 (0.66-1.96)	
